# Anti-Obesity Effects of a Mixture of *Atractylodes macrocephala* and *Amomum villosum* Extracts on 3T3-L1 Adipocytes and High-Fat Diet-Induced Obesity in Mice

**DOI:** 10.3390/molecules27030906

**Published:** 2022-01-28

**Authors:** Hae-Lim Kim, Sung-Kwon Lee, Da-Eun Min, Bong-Keun Choi, Dong-Ryung Lee

**Affiliations:** Research Institute, NUON Co., Ltd., Jungwon-gu, Seongnam 13201, Korea; ics1357@naver.com (H.-L.K.); sklee@nuon.kr (S.-K.L.); demin@nuon.kr (D.-E.M.); cbcbcbk@nuon.kr (B.-K.C.)

**Keywords:** *Atractylodes macrocephala*, *Amomum villosum*, anti-obesity, adipocytes, high-fat diet-induced obesity

## Abstract

Since the potential of (3:1) mixtures of *Atractylodes macrocephala* and *Amomum villosum* extracts has been proposed in the management of obesity, the purpose of present study was to investigate the effects of AME:AVE (3:1) mixture on weight loss, obesity-related biochemical parameters, adipogenesis and lipogenesis related proteins in 3T3-L1 cells and HFD-induced obesity in a mouse model. Treatment with AME:AVE (3:1) mixture inhibited lipid accumulation. Furthermore, the treatment with 75 and 150 mg/kg of AME:AVE (3:1) significantly decreased the body weight gain, white adipose tissue (WAT) weight, and plasma glucose level in HFD-induced obese mice. Moreover, treatment with 75 and 150 mg/kg AME:AVE (3:1) also significantly lowered the size of adipocytes in adipose tissue and reduced the lipid accumulation in liver. AME:AVE (3:1) treatment significantly decreased the expression of proteins related to adipogenesis and lipogenesis in 3T3-L1 adipocytes and WAT of HFD-induced obese mice. These results suggest that the AME:AVE herbal mixture (3:1) has anti-obesity effects, which may be elicited by regulating the expression of adipogenesis and lipogenesis-related proteins in adipocytes and WAT in HFD-induced obesity in mice.

## 1. Introduction

Obesity has become a significant clinical problem in recent decades due to its role in type 2 diabetes, hypertension, hyperlipidemia, and cardiovascular disease [1,2]. It is characterized by increased fat mass, which is associated with increased cell number (hyperplasia) and size (hypertrophy) [3,4]. For this reason, obesity has become a serious public health concern worldwide [5].

Adipocytes are important cells of fatty tissues. Excessive amounts of lipid (triglycerides) can accumulate in adipose tissues accompanied by increased levels of adipogenesis and lipogenesis, which leads to enhanced body weight gain [6]. Obesity and related diseases are strongly linked to adipocyte differentiation and fat accumulation [7]. 3T3-L1 cells are widely used in anti-obesity studies to investigate key molecular markers of adipocyte differentiation [8,9,10].

The different pharmacological approaches to prevent and treat obesity have several limitations such as undesirable and adverse effects [11,12]. Public attention is increasingly focused on using natural products to prevent and treat obesity with various plants such as fruits, vegetables, and herbs, with minimal side effects [13].

*Atractylodes macrocephala* is a medicinal herb that has been widely used in Chinese traditional medicine for approximately 2000 years [14,15]. It is rich in bioactive compounds such as atractylenolides I, II, and III [16,17] and exhibits various pharmacological properties, including antioxidant [18], anti-inflammatory [19], antimutation [20], and antitumor effects [21].

*Amomum villosum* Lour. (Zingiberaceae, a legal source of Amomi Fructus) is a classic traditional Chinese herb [22]. Pharmacological studies have shown that Amomi Fructus has strong anti-ulcer, anti-diarrhea, anti-inflammatory, and antimicrobial activities [23]. Recently, it has been reported that a mixture of *Atractylodes macrocephala* and *Amomum villosum* extracts significantly inhibited OP9 adipocyte differentiation [24] and weight gain induced by high-fat diet [25,26]. Especially, Kim et al. [26] reported that the anti-obesity effects mediated by decreased expression of adipogenesis- and lipogenesis-related genes expressions was confirmed in the diet-induced obesity 1 (DIO1) model induced with AME:AVE (3:1). Based on these studies, we hypothesized that AME:AVE (3:1) might also have anti-obesity effects in 3T3-L1 cells and obesity-induced DIO2 models.

Therefore, in this study, the anti-obesity effects and the underlying mechanisms of adipogenesis and lipogenesis in 3T3-L1 cells and HFD-induced obesity in mice model were investigated using a 3:1 mixture of *Atractylodes macrocephala* and *Amomum villosum* extracts reported by Kim et al. [24,25,26].

## 2. Results

### 2.1. Cytotoxic Effects of the Mixture of Atractylodes macrocephala and Amomum villosum Extracts (3:1) in 3T3-L1 Preadipocytes

**Figure 1:**
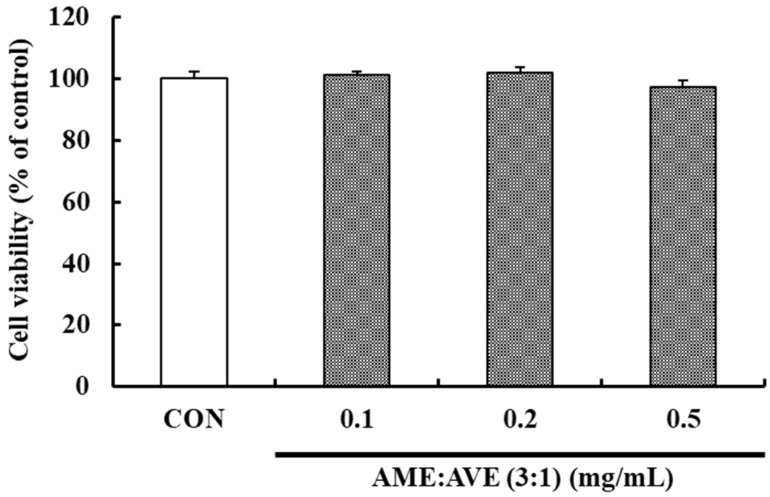
Cytotoxic effects of the mixture of *Atractylodes macrocephala* and *Amomum villosum* extracts (3:1) in 3T3-L1 preadipocytes. No significant difference was found in viability compared with the control group not treated with the herbal mixture (0.1, 0.2, and 0.5 mg/mL). Data represent mean ± SD of three independent experiments. CON, only dimethyl sulfoxide (DMSO) treated control.

The cytotoxic effect of AME:AVE (3:1) on the viability of 3T3-L1 preadipocytes was evaluated using the MTT assay. AME:AVE (3:1) did not exhibit cytotoxicity at any of the concentrations investigated (0.1, 0.2, and 0.5 mg/mL, Figure 1).

### 2.2. Effects of Mixtures of Atractylodes macrocephala and Amomum villosum Extracts (3:1) on Adipocyte Differentiation in 3T3-L1 Preadipocytes

**Figure 2:**
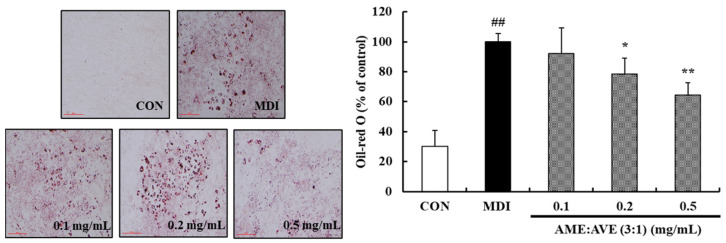
Effects of *Atractylodes macrocephala* and *Amomum villosum* extracts (3:1) on adipocyte differentiation in 3T3-L1 preadipocytes. Confluent 3T3-L1 preadipocytes were differentiated into adipocytes in media supplemented with or without AME:AVE (3:1) for 6 days. Lipid accumulation was measured via Oil-Red O staining and absorbance. Data represent mean ± SD of three independent experiments. Scale bar = 500 μm. CON, only dimethyl sulfoxide (DMSO) treated no differentiation control; MDI, only 3-isobutyl-1-methylxanthine, dexamethasone, and insulin treated differentiation control. ## *p* < 0.01 vs. CON group. * *p* < 0.05, and ** *p* < 0.01 vs. MDI group.

To investigate the effects of AME:AVE (3:1) mixture on adipocyte differentiation, 3T3-L1 adipocytes were treated with various concentrations of AME:AVE (3:1) (0.1, 0.2, and 0.5 mg/mL). Lipid concentrations in lipid droplets were tested with Oil-red O staining. The results show that mixtures of AME:AVE (3:1) decreased the lipid accumulation in 3T3-L1 adipocytes without cytotoxicity. Lipid accumulation was decreased by 23.57% (*p* < 0.05) and 67.12% (*p* < 0.01), respectively, at concentrations of AME:AVE (3:1) 0.2 and 0.5 mg/mL (Figure 2).

### 2.3. Effects of Mixtures of Atractylodes macrocephala and Amomum villosum Extracts (3:1) on Proteins in Adipogenesis and Lipogenesis Downstream in 3T3-L1 Adipocytes

Expression of key transcriptional factors involved in adipogenesis, such as PPARγ and C/EBPα, and transcriptional factor target genes, such as LPL and ap2, was also examined by western blotting. The results showed that levels of PPARγ, C/EBPα, LPL, and ap2 protein in MDI-induced 3T3-L1 adipocytes increased significantly compared with the control. However, treatment with mixtures of AME:AVE (3:1) at 0.1, 0.2, and 0.5 mg/mL inhibited the expression of PPARγ by 46.18, 60.35, and 66.73% (*p* < 0.01) and C/EBPα by 5.02, 19.99, and 64.49% (*p* < 0.01), respectively, and LPL in the range of 10.70–48.66% (*p* < 0.01) and ap2 in the range of 26.70–77.54% in a dose-dependent manner (*p* < 0.01).

**Figure 3:**
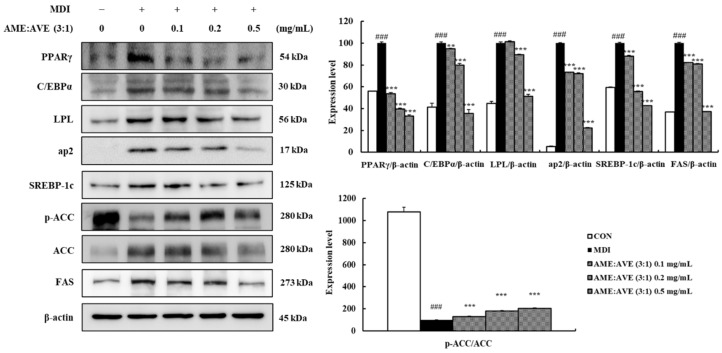
Effects of *Atractylodes macrocephala* and *Amomum villosum* extracts (3:1) on levels of downstream proteins associated with adipogenesis and lipogenesis in 3T3-L1 adipocytes. Western blot bands were analyzed using Image J software. β-actin was used as a control for normalization. Data represent mean ± SD of three independent experiments. CON, only dimethyl sulfoxide (DMSO) treated no differentiation control; MDI, only 3-isobutyl-1-methylxanthine, dexamethasone, and insulin treated differentiation control. ### *p* < 0.001 vs. CON group. ** *p* < 0.01, and *** *p* < 0.001 vs. MDI group.

To determine the mechanism of inhibition of lipid accumulation by mixtures of AME:AVE (3:1), we analyzed the expression of FAS. AME:AVE (3:1) treatment inhibited the expression of FAS dose-dependently compared with fully differentiated adipocytes where the percentage of inhibition ranged between 17.86% and 62.84% (*p* < 0.01). In addition, we investigated the expression of SREBP-1c, which is an important factor regulating lipid accumulation in adipocytes mediated via regulation of the expression of FAS, a lipogenic enzyme. The results showed that the mixtures of AME:AVE (3:1) suppressed the protein expression of SREBP-1c dose-dependently with an inhibition range of 12.06–57.30% (*p* < 0.01). ACC is a rate-limiting enzyme in the fatty acid synthesis pathway mediated via phosphorylation and inactivation. Phosphorylation of ACC was increased significantly (*p* < 0.01) (Figure 3).

### 2.4. Effects of Atractylodes macrocephala and Amomum villosum Extracts (3:1) on Upstream Proteins in Adipogenesis and Lipogenesis in 3T3-L1 Adipocytes

**Figure 4:**
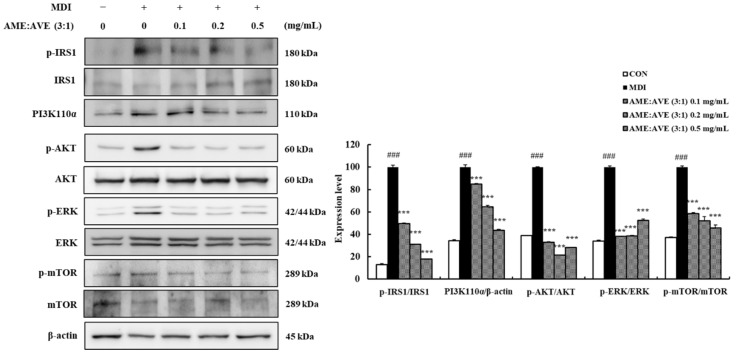
Effects of treatment with *Atractylodes macrocephala* and *Amomum villosum* extracts (3:1) on upstream expression of proteins related to adipogenesis and lipogenesis in 3T3-L1 adipocytes. Western blot bands were analyzed using Image J software. β-actin and total-form was used as a control for normalization. Data represent mean ± SD of three independent experiments. CON, only dimethyl sulfoxide (DMSO) treated no differentiation control; MDI, only 3-isobutyl-1-methylxanthine, dexamethasone, and insulin treated differentiation control. ### *p* < 0.001 vs. CON group. *** *p* < 0.001 vs. MDI group.

To further investigate the mechanism of AME:AVE (3:1) in adipogenesis and lipogenesis, the expression of p-IRS1, PI3K110α, p-AKT, p-ERK, and p-mTOR was measured. MDI-induced 3T3-L1 adipocytes significantly increased the expression of activated p-IRS1, PI3K110α, p-AKT, p-ERK and p-mTOR compared with the control group. However, AME:AVE (3:1) concentrations of 0.1, 0.2, and 0.5 mg/mL markedly decreased the expression of p-IRS1, PI3K, p-AKT, p-ERK, and p-mTOR (*p* < 0.01) (Figure 4).

### 2.5. Effects of Atractylodes macrocephala and Amomum villosum Extracts (3:1) on Body and Organ Weights of High-Fat Diet-Induced Obesity in Mice

**Figure 5:**
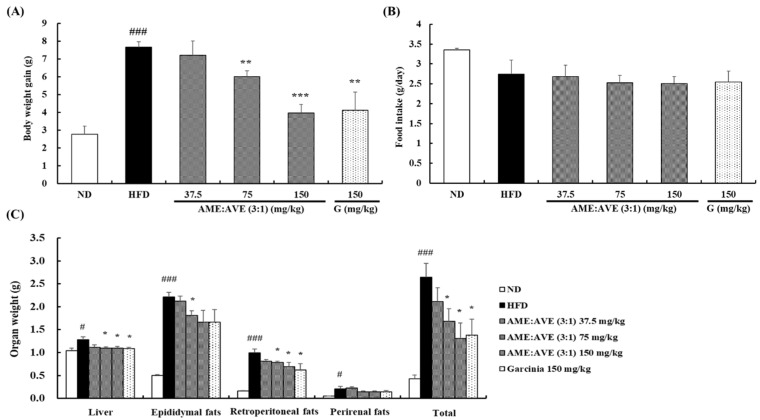
Effects of *Atractylodes macrocephala* and *Amomum villosum* extracts (3:1) on body and organ weights in high-fat diet-induced obesity in mice. (**A**) Body weight gain. (**B**) Food intake. (**C**) Organ weight. ND, normal-diet group; HFD, high-fat diet group. # *p* < 0.05, and ### *p* < 0.001 vs. ND group. * *p* < 0.05, ** *p* < 0.01, and *** *p* < 0.001 vs. HFD group.

Mixtures of AME:AVE (3:1) inhibited adipogenesis/lipogenesis in 3T3-L1 cells. Based on these results, we investigated the effect of AME:AVE (3:1) mixture on obesity in HFD-induced obesity in mice. The body weight gain of the HFD group was signifificantly increased when compared with the ND group after 8 weeks of feeding. In contrast, administration of AME:AVE (3:1) mixtures 75 and 150 mg/kg resulted in a significant decrease in body weight gain of 6.00 ± 0.34 g, and 3.96 ± 0.46 g (*p* < 0.01), respectively. (Figure 5A). Food intake did not display signifificant group wise differences (Figure 5B).

The weight of liver, total fat, and retroperitoneal fat showed a significant decrease (*p* < 0.05) in the AME:AVE (3:1) 75 and 150 groups compared with the HFD group. The weight of epididymal and perirenal fat increased significantly in the HFD group compared with the ND group, and a tendency toward decrease was detected following exposure to AME:AVE (3:1); however, the epididymal fat showed significance only in the AME:AVE (3:1) 75 group (*p* < 0.05) (Figure 5C).

### 2.6. Effects of Mixtures of Atractylodes macrocephala and Amomum villosum Extracts (3:1) on Liver and Epididymal Fat Morphology in High-Fat Diet-Induced Obesity in Mice

**Figure 6:**
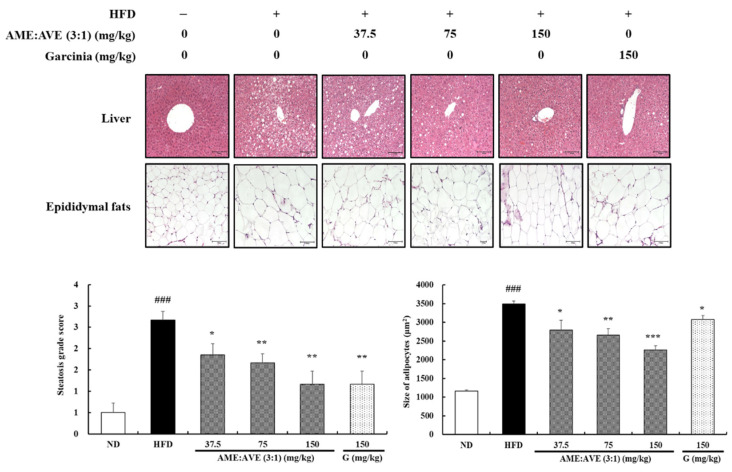
Effects of *Atractylodes macrocephala* and *Amomum villosum* extracts (3:1) on liver and epididymal fat morphology in high-fat diet-induced obesity in mice. Liver and epididymal adipose tissues were stained with hematoxylin and eosin and observed under a microscope. Steatosis grade scores of liver sections and the size of adipocyte area in epididymal fats were analyzed graphically. Scale bar = 100 μm. ND, normal-diet group; HFD, high-fat diet group. ### *p* < 0.001 vs. ND group. * *p* < 0.05, ** *p* < 0.01 and *** *p* < 0.001 vs. HFD group.

Histological observations of liver and epididymal fats were based on 6 groups: ND; HFD; HFD + AME:AVE (3:1) 37.5, 75, and 150; HFD + G 150 groups. Sections of liver and epididymal fats were stained with H&E to analyze the lipid accumulation and the cellular morphology. As a result, it was confirmed that administration of AME:AVE (3:1) decreased hepatic steatosis, in a dose-dependent manner (Figure 6).

The adipocyte size of epididymal fat in the HFD group showed an increase compared with the ND group. In contrast, the adipocyte size in the HFD + AME:AVE (3:1) groups showed a significant decrease compared with the HFD group. In particular, the AME:AVE (3:1) 150 group showed excellent reduction in adipocyte size (Figure 6).

### 2.7. Effects of Atractylodes macrocephala and Amomum villosum Extracts (3:1) on Plasma Biochemical Parameters in High-Fat Diet-Induced Obesity in Mice

The plasma biochemical parameters, including glucose, T-CHO, TG, LDL-c, HDL-c/LDL-c ratio, ALT, AST, leptin, and adiponectin were confirmed. As shown in Table 1, glucose, T-CHO, TG, LDL-c, ALT, AST, and leptin levels were markedly increased and HDL-c/LDL-c ratio and adiponectin levels were decreased in the HFD group. However, the glucose levels in AME:AVE (3:1) groups were significantly decreased in a dose-dependent manner (*p* < 0.01). In addition, ALT and AST levels of AME:AVE (3:1) 75 and 150 groups were also significantly decreased (*p* < 0.05). T-CHO, TG, LDL-c, and leptin levels showed a tendency to decrease in the group treated with AME:AVE (3:1), whereas T-CHO and leptin levels showed significance only in the AME:AVE (3:1) 75 group. The HDL-c/LDL-c ratio and adiponectin levels tended to increase in the group exposed to AME:AVE (3:1).

### 2.8. Effects of Mixtures of Atractylodes macrocephala and Amomum villosum Extracts (3:1) on Adipogenesis- and Lipogenesis-Related Protein Levels in High-Fat Diet-Induced Obesity in Mice

**Figure 7:**
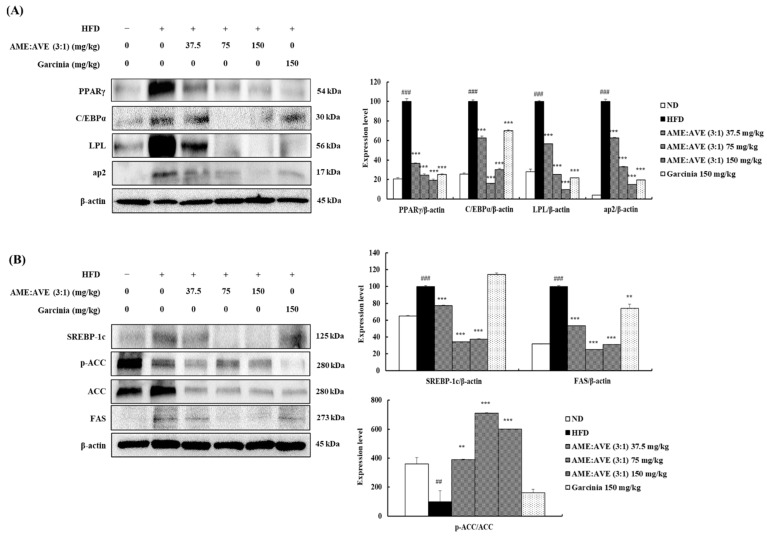
Effects of exposure to *Atractylodes macrocephala* and *Amomum villosum* extracts (3:1) on proteins related to adipogenesis and lipogenesis in high-fat diet-induced obesity in mice. (**A**) Adipogenesis-related protein levels. (**B**) Lipogenesis-related protein levels. Western blot bands were analyzed using Image J software. β-actin was used as a control for normalization. Data represent mean ± SD of three independent experiments. ND, normal-diet group; HFD, high-fat diet group. ## *p* < 0.01, and ### *p* < 0.001 vs. ND group. ** *p* < 0.01, and *** *p* < 0.001 vs. HFD group.

Western blot analysis was used to confirm the levels of adipogenesis- and lipogenesis-related proteins. HFD group showed a significant increase in PPARγ, C/EBPα, LPL, and ap2 protein expression in epididymal fat. In contrast, treated with AME:AVE (3:1) significantly decreased the expression of PPARγ and C/EBPα PPARγ, C/EBPα, LPL, and ap2 in epididymal fat (*p* < 0.01) (Figure 7A). As shown in Figure 7B, the levels of SREBP-1c, ACC, and FAS protein were increased, while p-ACC level was decreased in the HFD group. Our results revealed that AME:AVE (3:1) treatment markedly inhibited this increase in epididymal fat and also increased the p-ACC level significantly (*p* < 0.01) (Figure 7B).

## 3. Discussion

According to the report of Song et al. [27], the extract of *Atractylodes macrocephala* significantly reduced the weight gain induced by a high-fat diet, and this effect was related to a reduction in hepatic fat accumulation. In addition, Lu et al. [28] reported that *Amomum villosum* inhibits increased fatty liver formation mediated via gut-liver axis.

Recently, the treatment with *Atractylodes macrocephala* and *Amomum villosum* extract (AME:AVE (3:1)) resulted in excellent anti-obesity effect [25]. The mixture at a ratio of 3:1 significantly inhibited OP9 adipocyte differentiation [24] and weight gain induced by high-fat diet [25,26]. In particular, Kim et al. [26] reported that the anti-obesity effects was confirmed in the DIO1 model administrated with AME:AVE (3:1) while inducing obesity. Based on these studies, the present study confirmed the anti-obesity effects and the underlying mechanisms of adipogenesis and lipogenesis in 3T3-L1 cells. Then, we confirmed the anti-obesity effects in the DIO2 model in which the AME:AVE (3:1) was administered with mice after inducing obesity.

Our results suggested that AME:AVE (3:1) treatment inhibited the differentiation and lipid accumulation of 3T3-L1 adipocytes. This result was further supported by in vivo experiments, showing that AME:AVE (3:1) treatment for 8 weeks significantly reduced the weight gain, and the weight of liver and retroperitoneal fats in HFD-induced obesity in mice. Additionally, AME:AVE (3:1) treatment did not affect the food intake in HFD-induced obesity in mice, indicating that the weight-loss effect of AME:AVE (3:1) was not due to a reduction in food intake. These results are consistent with previous reports suggesting that the mixture of AME:AVE (3:1) has anti-obesity effects.

Obesity is closely related to hyperplasia and hypertrophy of adipose cells [29,30] Adipocyte hypertrophy is caused by excessive accumulation of fat within the cells due to excessive energy intake. Since adipocyte proliferation occurs due to the complex interaction between proliferation and differentiation of preadipocytes [31], the 3T3-L1 preadipocyte MDI-induced differentiation system was utilized in this experiment. We demonstrated that AME:AVE (3:1) treatment suppressed lipid accumulation during 3T3-L1 adipocyte differentiation without cytotoxicity. Also, the treatment of HFD-fed mice with AME:AVE (3:1) resulted in a significant decrease in liver and total fat weight. In addition, histological data showed a decrease in adipocyte size in liver and epididymal fat in AME:AVE (3:1) groups, suggesting effects on lipid accumulation and adipocyte size in adipose tissue, which is likely related to reduced fat weight.

In obesity, fatty liver is induced by the accumulation of fat in the liver tissue [32]. Histological data of the liver showed that AME:AVE (3:1) treatment reduced the size and number of lipid droplets and restored tissue structure, which indicates that AME:AVE (3:1) treatment inhibits lipid accumulation in the liver. In our study, AME:AVE (3:1) treatment lowered the levels of plasma glucose and T-CHO and also tended to decrease TG and LDL-c levels. These results suggest that AME:AVE (3:1) treatment ameliorates hyperlipidemia in HFD-induced obesity in mice by reducing hepatic fat accumulation.

Excessive fat intake triggers oxidative stress, which leads to hepatic dysfunction and fatty degeneration via abnormal activation of ALT and AST [33]. In this study, AME:AVE (3:1) treatment reduced plasma ALT and AST levels in HFD-induced obesity in mice compared with the HFD group, and thereby ameliorated liver dysfunction induced by fat accumulation.

In obesity, the accumulation of visceral fat affects adipokines in adipose tissue including leptin and adiponectin. Leptin promotes energy consumption by inducing satiety in the central circuits of the hypothalamus, and adiponectin increases fat oxidation and inhibits lipid accumulation. It is known that the concentration of leptin increases and the concentration of adiponectin decreases in obesity [34]. Therefore, the concentrations of leptin and adiponectin in the serum were measured to evaluate the effect of exposure to AME:AVE (3:1) on serum adipokines in mice. Leptin showed a significant decrease in AME:AVE (3:1) 75 group and adiponectin levels showed a tendency to increase in the group treated with the herbal extracts.

To investigate the underlying anti-obesity mechanism associated with AME:AVE (3:1), we measured the levels of lipogenesis- and adipogenesis-related proteins in 3T3-L1 adipocytes and epididymal fats in HFD-induced obesity in mice.

Adipogenesis, which is indicated by preadipocyte differentiation into mature adipocytes, is causally linked to obesity. Regulation of adipocyte differentiation genes and proteins controls adipogenesis. Insulin signaling is a key signal underlying adipogenesis regulation mediated via activation of glucose uptake and inhibition of lipolysis in adipocytes [35,36]. IRS-1 belongs to a family of docking molecules that link the activation of IR, which activate downstream kinase cascades including PI3K and AKT [37]. Activation of IRS-1 is mediated via phosphorylation of serine residues and enhanced IRS-1-related PI3K activity [38]. In addition, insulin signaling is intimately linked to the nutrient-responsive mammalian target of rapamycin (mTOR) signaling pathway via activation of AKT [39]. Phosphorylation of IRS-1 and AKT, ERK, and mTOR was enhanced in 3T3-L1 adipocytes but inhibited by AME:AVE (3:1) treatment.

PPARγ is an important factor regulating adipocyte differentiation and adipogenesis. C/EBPα promotes adipocyte differentiation via interaction with PPARγ, which increases the expression of adipocyte-specific genes such as aP2 and LPL [40,41]. SREBP-1c is also a transcription factor that plays an important role in lipid metabolism by increasing the expression of several adipogenic genes, such as FAS, in adipose tissue and liver. PPARγ is activated via regulation of expression and production of endogenous PPARγ ligands [42,43]. Lipoprotein lipase is a major regulator of blood TG levels via breakdown of lipoprotein triglycerides. It plays an important role in transporting fat and hydrolyzed fat transporting lipoproteins [44]. The binding of these transcription factors triggers the expression of target genes and proteins responsible for TG synthesis and storage, leading to accelerated adipogenesis. ACC is a rate-limiting enzyme in the fatty acid synthesis via phosphorylation and inactivation [45]. We found that treatment with AME:AVE (3:1) reduced the expression of PPARγ, C/EBPα, aP2, and FAS proteins in 3T3-L1 adipocytes, and increased ACC phosphorylation. The same factors showed significant results following treatment with AME:AVE (3:1) in the epididymal fat of high-fat diet-induced mice. Our results suggest that AME:AVE (3:1) treatment modulates the insulin signaling pathway and suppresses the expression of adipogenesis and lipogenesis-related proteins, thereby resulting in anti-obesity effects.

**Figure 8:**
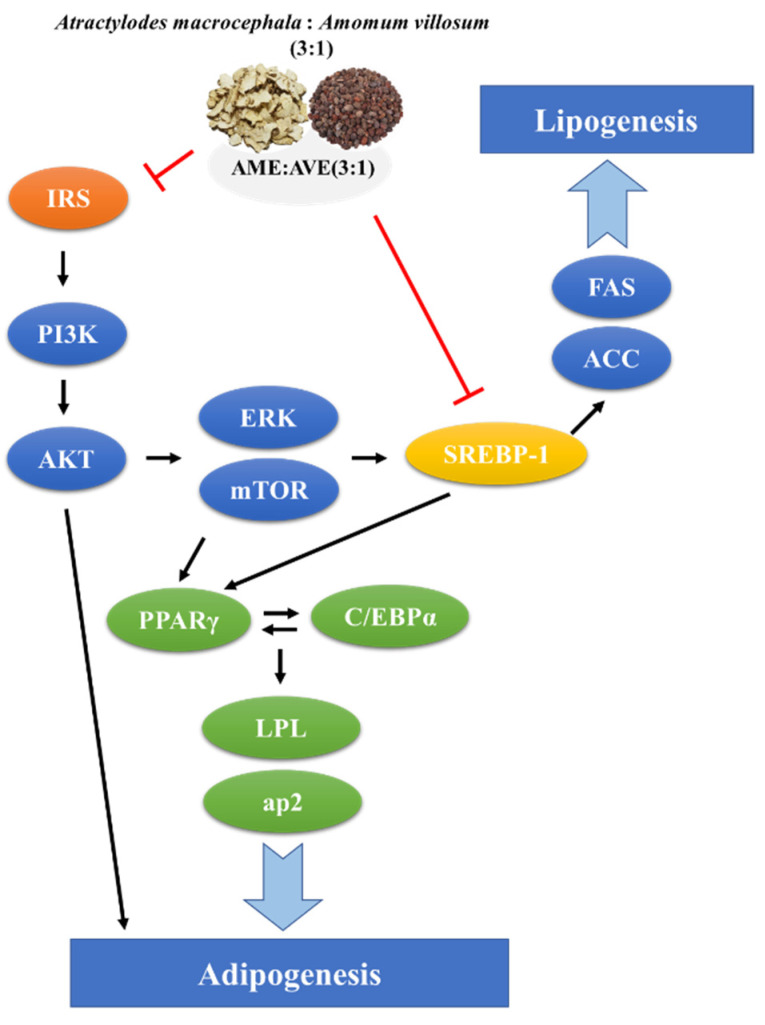
The proposed anti-obesity effects mechanism of *Atractylodes macrocephala* and *Amomum villosum* extracts (3:1).

In conclusion, our results showed that AME:AVE (3:1) treatment inhibited lipid accumulation in 3T3-L1 adipocytes, and reduced body weight gain, organ weight, adipocyte size, and regulated plasma biochemical levels in the HFD-mice model. In addition, it regulated the proteins related to adipogenesis and lipogenesis in 3T3-L1 adipocytes and HFD-induced obesity in mice, and the insulin regulatory pathway was confirmed in 3T3-L1 adipocytes (Figure 8). These findings indicated that AME:AVE (3:1) prevents the development of obesity and hyperlipidemia in 3T3-L1 adipocytes and HFD-induced obesity in mice, which provide valuable insight into the potential benefits of AME:AVE (3:1) in obesity. Moreover, it is considered that additional clinical studies are needed to suggest the possibility of application in the prevention of obesity and obesity-related diseases in humans.

## 4. Materials and Methods

### 4.1. Chemicals and Reagents

3-Isobutyl-1-methylxanthine (IBMX), dexamethasone, and insulin were obtained from Wako Pure Chemical Industries Ltd. (Osaka, Japan). Dulbecco’s modified Eagle’s medium (DMEM), bovine calf serum (BCS), and penicillin/streptomycin were purchased from Gibco BRL (Grand Island, NY, USA). Fetal bovine serum (FBS) was obtained from ATLAS Biologicals (Fort Collins, CO, USA). The following antibodies were ordered from Santa Cruz Biotechnology (Dallas, TX, USA): Peroxisome proliferator-activated receptor γ (PPARγ; #sc-7273), CCAAT/enhancer-binding protein α (C/EBPα; #sc-365318), lipoprotein lipase (LPL; #sc-373759), sterol regulatory element binding proteins (SREBP-1c; #sc-365513). Adipocyte fatty acid-binding protein 2 (ap2; #PA5-30591) was acquired from Invitrogen (Carlsbad, CA, USA). β-actin (#8457), phospho-acetyl-CoA carboxylase (p-ACC; #3661), ACC (#3676), fatty acid synthase (FAS; #3180), phospho-insulin receptor substrate 1 (p-IRS1; #2388), IRS1 (#2382), phosphoinositide 3-kinase 110α (PI3K110α; #4249), phospho-protein kinase B (p-AKT; #4060), AKT (#4691), phospho-extracellular signal-regulated kinase (p-ERK; #4377), ERK (#4695), phospho-mechanistic target of rapamycin (p-mTOR; #2974), mTOR (#2972) were obtained from Cell Signaling Technology (Danvers, MA, USA). Horseradish peroxidase (HRP)-linked anti-rabbit IgG (#SA002-500) and HRP-linked anti-mouse IgG (#SA001-500) were purchased from GenDEPOT (Barker, TX, USA).

### 4.2. Preparation of AME:AVE (3:1) Extracts

AME:AVE (3:1) is a mixture of the aqueous extracts of two herbs, namely *Atractylodes macrocephala* and *Amomum villosum*, in a 3:1 ratio. The AME:AVE (3:1) mixture manufacturing process was as follows: *Atractylodes macrocephala* and *Amomum villosum* were extracted using water. The resulting solutions were concentrated and dried to yield AME and AVE, respectively. Then, the mixture it is prepared by mixing AME and AVE in a ratio of 3:1. This manufacturing process was designed and developed by NUON Co., Ltd., (Seongnam, Korea), who manufactures and sells this product. To confirm the composition of AME:AVE (3:1) mixture, we performed high-performance liquid chromatography (HPLC) analysis. Atractylenolide III is a phytochemical marker for *Atractylodes macrocephala* raw material, while vanillic acid is phytochemical marker for *Amomum villosum* raw material, respectively. For in vitro studies, AME:AVE (3:1) was dissolved in dimethyl sulfoxide (DMSO) at concentration of 100, 200, and 500 mg/mL, respectively and then diluted in culture medium at concentration of 0.1, 0.2, and 0.5 mg/mL. respectively. Control is 3T3-L1 cells treated with culture medium containing only DMSO (final DMSO concentration 0.1%). For in vivo studies, AME:AVE (3:1) extracts were homogenized in 0.5 mL 0.5% carboxymethyl cellulose sodium salt (CMC-Na) at concentration of 37.5, 75, and 150 mg/kg, respectively and orally administered. The control mice group were given the same volume of 0.5% CMC-Na instead of the test solution.

### 4.3. Cell Culture

3T3-L1 preadipocytes (American Type Culture Collection, Rockville, MD, USA) cells were cultured in DMEM with 10% bovine calf serum (BCS), 1% penicillin/streptomycin and maintained in a humified atmosphere of 5% CO_2_ at 37 °C. To induce adipocyte differentiation, fully confluent preadipocytes were treated with MDI (3-isobutyl-1-methylxanthine, dexamethasone, and insulin) differentiation medium (DMEM, 10% fetal bovine serum (FBS), 0.5 mM IBMX, 5 μg/mL insulin, and 1 μM dexamethasone) with or without AME:AVE (3:1) (defined as Day 0). After two days of incubation, the cell medium was replaced by DMEM supplemented with 10% FBS and 5 μg/mL of insulin in the presence or absence of AME:AVE (3:1) (Day 2). After another two days, the medium was changed with DMEM containing 10% FBS (Day 4). 3T3-L1 preadipocytes were completely differentiated to mature adipocytes on Day 6.

### 4.4. MTT Assay for Cell Viability

3T3-L1 preadipocytes were seeded on 96-well plates and treated with various concentration of AME:AVE (3:1) for 24 h. Cells treated with 3-(4,5-dimethylthiazol-2-yl)-2,5-diphenyl tetrazolium bromide (MTT) solution (5 mg/mL) were incubated at 37 °C for 4 h. After removal of the supernatant, 100 μL of DMSO was added to dissolve formazan crystals, and the MTT-formazan product was measured using a microplate plate reader (Tecan, Mannedorf, Switzerland).

### 4.5. Oil-Red O Staining

Differentiated 3T3-L1 adipocytes were washed three times with PBS and fixed with 10% (*v*/*v*) formalin solution (Sigma-Aldrich, St. Louis, MO, USA) for 1 h. Fresh Oil-red O stock solution was mixed with distilled water in a ratio of 6:4 to obtain the working solution, followed by incubation for 1 h at room temperature (RT), and filtered. After fixation, cells were washed three times with distilled water and then stained with Oil-red O working solution at 25 °C for 2 h. Cells were rinsed three times with distilled water and photographed with an ECLIPSE Ts2 microscope (Nikon Corporation, Tokyo, Japan). The Oil-red O dye was eluted with isopropanol to determine the intracellular lipid content using a microplate reader (Tecan) at 520 nm.

### 4.6. Animals and Diets

This experiment was carried out in compliance with the Animal Experimental Ethics Regulations of INVIVO Co., Ltd. (Chungnam, Korea) (Approval No. IV-RA-04-2010-36). Experimental animals were acquired from Orient Bio Co., Ltd (Seongnam, Korea). Five-week-old male C57BL/6 mice (*n* = 48) in specific-pathogen free (SPF) state were used in the experiment after a week of acclimatization. The experimental diet during the acclimatization period included general solid feed (Purina Lab Rodent Chow #38057, Purina Co., Seoul Korea). The mice were housed in a controlled atmosphere (23 ± 1 °C at 50 ± 5% relative humidity) with a 12 h light/dark cycle. Feed and water were provided ad libitum.

After acclimatization, the mice were fed either the normal-fat diet (ND, *n* = 8) as a normal group or high-fat diet (HFD, *n* = 40, rodent diet with 60% kcal fat (#D12492, Research diets INC) as the HFD induced obese group for 5 weeks. The obesity animal model when the weight gain was about 20% compared with the normal group, the obesity was established. After obesity induction, the mice with obesity were further divided into 5 experimental groups (*n* = 8/group) and were matched by body weight. The following groups were studied for 8 weeks: ND group; HFD group; AME:AVE (3:1) 37.5 group (high-fat diet + AME:AVE (3:1) 37.5 mg/kg); AME:AVE (3:1) 75 group (high-fat diet + AME:AVE (3:1) 75 mg/kg); AME:AVE (3:1) 150 group (high-fat diet + AME:AVE (3:1) 150 mg/kg). Another HFD group received oral administration of 150 mg/kg of *Garcinia cambogia* extract (G 150) daily as a positive control group because of the weight loss effects of *Garcinia cambogia* extract [46,47,48].

Weight, diet and water intake were recorded weekly. In order to observe the external changes before autopsy, bodies were photographed in each group, and the animals were anesthetized via inhalation anesthesia before blood was drawn. After sacrificing the mice, the tissues were excised. Before tissue excision, photographs were obtained to confirm the accumulation of adipose tissue in the abdomen, and liver, epididymal fats, retroperitoneal fats, and perirenal fats were extracted. The excised tissues were photographed to observe external changes, and the weight of each organ was measured. The tissues were then divided and fixed in 10% neutral formalin, and frozen. Frozen tissues were stored at −80 °C until use.

### 4.7. Biochemical Analysis

After the blood was coagulated for 30 min, the serum was recovered by centrifugation at 3000 rpm for 10 min. The levels of total cholesterol (T-CHO), triglyceride (TG), glucose, low density lipoprotein cholesterol (LDL-C), high density lipoprotein cholesterol (HDL-C), alanine aminotransferase (ALT), and aspartate aminotransferase (AST) in the recovered serum were analyzed. The contents of leptin and adiponectin in the blood were analyzed using an ELISA kit (leptin, ab199082, Abcam, Cambridge, UK; adiponectin, MRP300, R&D Systems, Wiesbaden, Germany).

### 4.8. Histological Analysis

The fixed liver and epididymal fats were paraffinized, cut into slices, attached to slides, and analyzed via hematoxylin & eosin staining. The stained slides were photographed under a microscope, and the fat tissue area was measured using the Image J program developed at the National Institutes of Health (Bethesda, MD, USA).

### 4.9. Protein Extraction and Western Blot Analysis

3T3-L1 adipocytes were lysed in CelLytic buffer (Sigma-Aldrich) and then centrifuged at 12,000 rpm for 20 min at 4 °C. Epididymal fat was washed with cold PBS and RIPA Homogenize buffer containing 50 mM Tris-HCl pH 7.4, 150 mM NaCl, 1 mM EDTA, 1% Triton X-100, 1% sodium deoxycholate, 0.1% SDS, 1 mM PMSE, and 1% protease inhibitor. The homogenate was also centrifuged at 12,000 rpm for 20 min at 4 °C. After the above procedure, both cells and adipose tissue supernatant were collected. Protein concentration was measured via Bradford assay (Bio-Rad Laboratories, Hercules, CA, USA). Proteins were separated via SDS-PAGE and transferred to Immobilon-P membranes (Millipore, Bedford, MA, USA) and then membranes were cut to detect the location of each biomarker specific according to each antibody manufacturer’s datasheet. The transferred membrane was blocked with 5% skim milk dissolved in Tris-buffered saline containing 0.1% Tween-20 (TBS-T) for 1 h, and then incubated overnight at 4 °C with a specific primary antibody. The blots were then incubated in the corresponding horseradish peroxidase-conjugated anti-rabbit or anti-mouse immunoglobulin G for 1 h at room temperature (RT). Detection was performed with ECL solution (GenDEPOT), and band intensity was measured using a LuminoGraph (Atto, Tokyo, Japan). The band images were digitized using the Image J program and corrected by dividing each group result value by each β-actin value and there was no significant difference between every β-actin-to-β-actin levels.

### 4.10. Statistical Analysis

All data are presented as mean ± standard deviation, and statistical calculations were performed using the *t*-test and ANOVA (one-way analysis of variance) with Bonferroni’s multiple comparison tests. *p* < 0.05, *p* < 0.01, and *p* < 0.001 were used to indicate statistical significance [49,50,51].

## Figures and Tables

**Table 1:** Effects of *Atractylodes macrocephala* and *Amomum villosum* extracts (3:1) on plasma biochemical levels in high-fat diet-induced obesity in mice.

Indexes	ND	HFD	HFD			
			AME:AVE (3:1) 37.5	AME:AVE (3:1) 75	AME:AVE (3:1) 150	G 150
Glucose (mg/dL)	251.0 ± 15.1	374.5 ± 13.9 ^###^	305.9 ± 9.8 **	325.3 ± 2.8 **	268.6 ± 6.0 ***	301.8 ± 8.7 **
T-CHO (mg/dL)	97.5 ± 3.7	205.5 ± 19.5 ^###^	186.0 ± 5.1	160.2 ± 3.1 *	159.5 ± 4.0	165.5 ± 4.9
TG (mg/dL)	73.0 ± 2.6	98.3 ± 5.3 ^##^	106.3 ± 3.2	92.5 ± 3.1	85.6 ± 4.4	83.5 ± 2.0 *
LDL-c (mg/dL)	9.4 ± 0.2	37.7 ± 7.0 ^##^	32.7 ± 0.8	29.3 ± 0.9	31.5 ± 1.3	31.9 ± 1.3
HDL-c/LDL-c	11.3 ± 0.5	4.7 ± 0.4 ^##^	4.9 ± 0.1	5.3 ± 0.2	4.9 ± 0.3	4.8 ± 0.3
ALT (U/L)	29.3 ± 3.9	50.6 ± 6.9 ^##^	40.6 ± 4.5	32.6 ± 1.4 *	32.7 ± 2.6 *	29.8 ± 0.9 *
AST (U/L)	129.4 ± 8.6	185.9 ± 20.2 ^#^	144.6 ± 9.3	140.2 ± 3.6 *	125.1 ± 8.5 *	134.7± 2.9 *
Leptin (ng/mL)	1.5 ± 0.1	48.8 ± 9.1 ^###^	35.4 ± 3.1	26.0 ± 1.4 *	26.7 ± 6.7	33.6 ± 3.7
Adiponectin (ng/mL)	14.2 ± 1.0	13.9 ± 0.5	14.1 ± 0.6	14.4 ± 1.1	14.7 ± 0.3	12.0 ± 0.3

ND, normal-diet group; HFD, high-fat diet group. ^#^ *p* < 0.05, ^##^ *p* < 0.01, and ^###^ *p* < 0.001 vs. ND group. * *p* < 0.05, ** *p* < 0.01, and *** *p* < 0.001 vs. HFD group.

## Data Availability

Not applicable.

## Sample Availability

Samples of the compounds are not available from the authors.
